# Maneuver protocol for outpatient telemetric intracranial pressure monitoring in hydrocephalus patients

**DOI:** 10.1007/s00381-022-05659-5

**Published:** 2022-09-13

**Authors:** Valentina Pennacchietti, Andreas Schaumann, Ulrich-Wilhelm Thomale

**Affiliations:** Pediatric Neurosurgery, Charité Universitaetsmedizin Berlin, Campus Virchow Klinikum, Augustenburger Platz 1, 13353 Berlin, Germany

**Keywords:** Telemetric intracranial pressure monitoring, Sensor reservoir, Ventriculo-peritoneal shunt, Hydrocephalus, Intracranial compliance

## Abstract

**Introduction:**

Telemetric intracranial pressure measurement (tICPM) offers new opportunities to acquire objective information in shunted and non-shunted patients. The sensor reservoir (SR) provides tICPM modality at a decent sampling rate as an integrated component of the CSF shunt system. The aim of this study is to perform tICPM during a defined protocol of maneuvers in an outpatient setting as feasibility study including either shunt-dependent patients or candidates for possible shunt therapy.

**Methods:**

A total of 17 patients received a SR and were investigated within a protocol of maneuver measurements involving different body postures (90°, 10°, 0°, and − 10°), breathing patterns (hypo- and hyperventilation), and mild venous congestion (Valsalva, Jugular vein compression), while the latter two were performed in lying postures (10° and 0°). The cohort included 11 shunted and 6 non-shunted (stand-alone-SR) patients. All measurements were evaluated using an ICP-analysis software (ICPicture, Miethke, Germany) looking at ICP changes and amplitude (AMP) characteristics.

**Results:**

The shunted patient group consisted of 11 patients (median age: 15.8 years; range: 4–35.2 years) with either a primary shunt (n=9) and 2 patients received a shunt after stand-alone-SR tICPM. Six patients were enrolled with a stand-alone SR (median age 11.9 years, range 3.6–17.7 years). In the stand-alone SR group, maneuver related ICP and AMP changes were more sensitive compared to shunted patients. Postural maneuvers caused significant ICP changes in all body positions in both groups. The highest ICP values were seen during Valsalva maneuver, provoked by the patients themselves. In the stand-alone group, significant higher ICP values during hyperventilation were observed compared to shunted individuals. In shunted patients, a significant correlation between ICP and AMP was observed only during hyperventilation maneuver, while this correlation was additionally seen in Valsalva and jugular vein compression in stand-alone patients.

**Conclusion:**

SR-related tICPM is helpful to objectify diagnostic evaluation in patients with CSF dynamic disturbances. The defined protocol did result in a wide range of ICP changes with promising potential for effective outpatient tICPM investigation. Since the correlation of ICP and AMP was observed during mild venous congestion maneuvers it appears to be specifically helpful for the evaluation of intracranial compliance. Further investigations of maneuver-related tICPM in a larger population, including variable pathologies, are needed to further establish the protocol in the clinical practice.

## Introduction

ICP measurement methods are usually conducted with direct invasive methods such as intraparenchymal probes or external ventricular drainages as performed during hospital care. Recently, telemetric tICPM was introduced, which enables the detection of intracranial pressure in a closed system to acquire single or continuous data also in an outpatient set-up [1, 2]. Different systems on the market offer the possibility to collect intracranial pressure data at different frequencies and with different modalities. Nowadays, one of the most frequently used telemetric systems is the SR (Miethke, Potsdam, Germany), which enables the direct integration in a shunt system to measure the ICP at a relatively high sampling rate (44 Hz) [3]. The SR has a given functional duration of 5 years as indicated by the manufacturer. However, previous reports have described a rather unlimited implantation time. This holds specifically true since the SR was designed to be included in a shunt system. The manufacturer indicates a possible drift of < 2 mmHg in 4 years. A few reports have described the advantage of the system to be used also as “stand-alone” diagnostic tool offering ICP measurements and direct access to CSF for possible volume relieve [4–7].

TICPM has opened the perspective for a new understanding of CSF pathophysiology. ICP can be evaluated not only in hospitalized patients in a lying position but could shed new light on the understanding of ICP characteristics during normal daily activities [8, 9]. Thus, it is largely contributing to better understand posture- or maneuver-related ICP changes. Studies about normal values of ICP have described the crucial role of the venous system, especially in the transition phase from lying to the upright body postures and vice versa [10–14]. Hereby, it was found that ICP values diminish, especially during body erection from 0° to 20°, and they remain rather stable at negative values toward 90° vertical body position, most likely due to the collapse of the jugular veins, inhibiting further intracranial blood volume loss [9]. It has also been widely accepted that negative ICP values are normal in the upright position to a certain degree [15]. Thereby, the regular normal ICP thresholds defined for hospitalized patients in lying positions are not valid during daily activity. Our previous experience in mostly shunted patients has defined a posture-related normal range of ≤ 10 cmH_2_O in a lying position and ≥ − 10 cmH_2_O in an upright position, however further validation will still be necessary and might largely differ in individual patient conditions [4, 6].

One major challenge of tICPM is the acquisition of relevant data and their post-processing, which needs to be efficiently planned for the time-restricted environment in clinical practice. For example, ICP home monitoring has been described, offering the possibility to associate event-related ICP changes in order to better understand the correlation between symptomatology and ICP in the individual patient during their daily routine [5]. However, the data acquired often exceeds the amount that can be easily analyzed in a reasonable time frame, thus new efforts are necessary to effectively work on big data sets. In outpatient practice, it has become obvious that the current condition of the patient in terms of symptoms and ICP condition can efficiently be evaluated using different posture maneuvers. We have found, in individual patients, that other maneuvers such as different breathing patterns or mildly induced venous congestion do also result in relevant changes in ICP values. Thus, the aim of this paper is to evaluate and define a maneuver protocol and its related ICP changes in an outpatient setting in order to gain a better understanding of the most relevant maneuvers applicable for therapeutic decision-making.

## Methods

### Measurement protocol

**Fig. 1:**
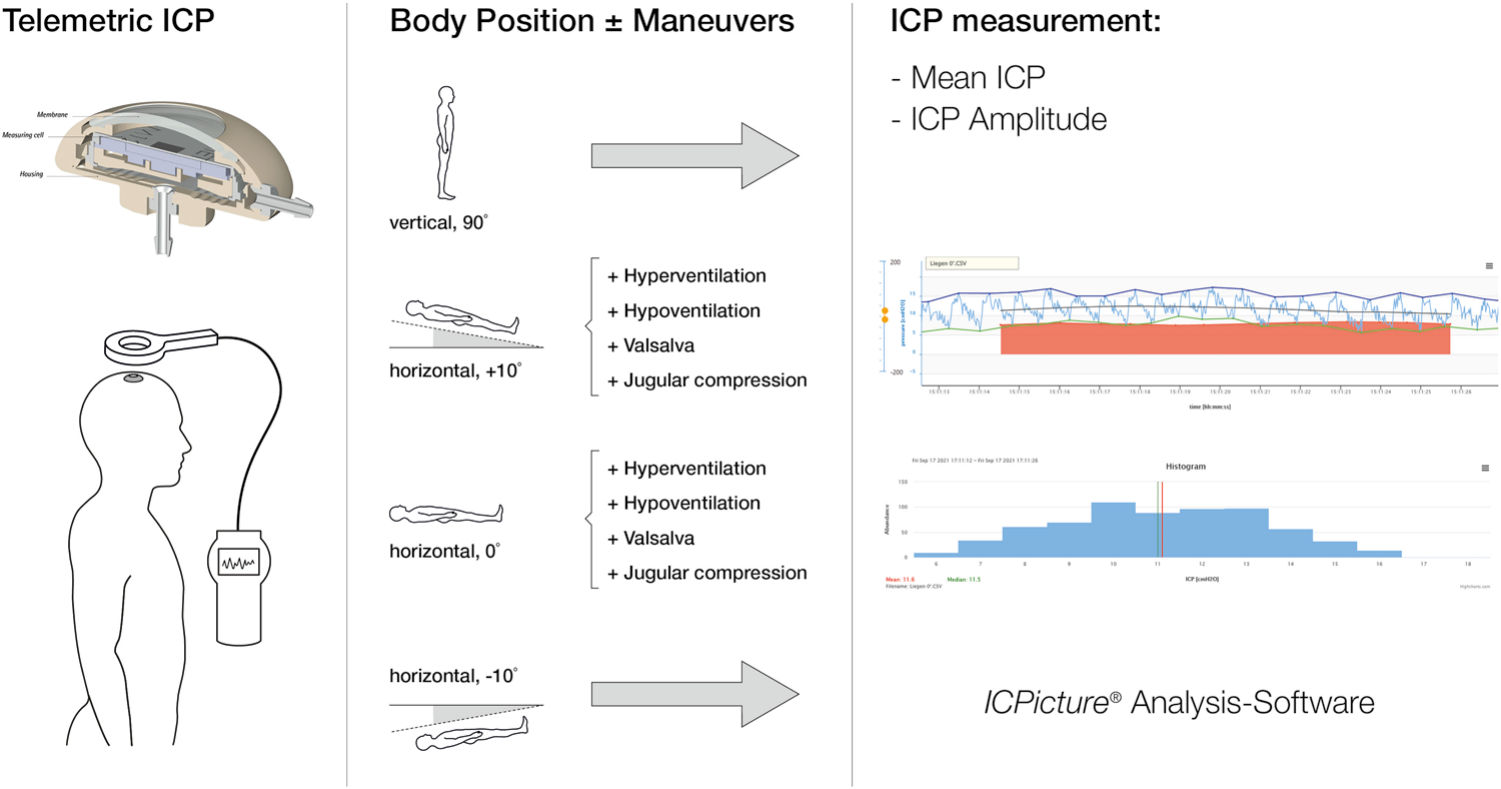
Outpatient maneuver protocol for telemetric intracranial pressure monitoring (tICPM). The Sensor Reservoir (SR) is subcutaneously implanted at a precoronal burr hole integrated into a shunt system or as a stand-alone implant. The trowel-connected monitor system allows noninvasive intracranial pressure (ICP) measurement (left column). The maneuver protocol includes postural body position alterations in standing and 10°, 0°, and − 10° lying positions. The lying positions (10° and 0°) are combined with additional ventilation (hyperventilation/hypoventilation) and venous congestion (Valsalva, jugular vein compression) maneuvers (middle column). ICP curve analysis was performed offline by using the software tool ICPicture (Miethlke, Potsdam, Germany) extracting the mean ICP values and the ICP pulse amplitude values extracted as mean from 6 curve cycles. In a histogram of ICP measurements, the range and frequency of measured ICP values from the maneuvers are given, respectively (right column)

The defined maneuver protocol included 12 different settings, which consist of postural changes, breathing patterns and induction of mild venous congestion. The positional maneuvers included the standing position (90°), lying supine in a + 10° anti-Trendelenburg position, lying supine in a 0° position and lying supine in a − 10° Trendelenburg position. The positional changes were performed using a specific patient bed with measurable angulation settings. Breathing and mild venous congestion maneuvers were applied both in + 10° supine, anti-Trendelenburg position and the 0° supine lying position. Breathing maneuvers included fast breathing (hyperventilation) and intermittent breath keeping (hypoventilation) for 30–60 s as tolerated without discomfort. Either Valsalva maneuver or mild external jugular vein compression at the neck provoked venous congestion and was applied for about 10–20 s as tolerated by the patients (Fig. 1). Patient comfort during all maneuvers was guaranteed throughout the time of measurement. Between each measurement, a break of at least 1 min was facilitated. The measurements were performed in the outpatient clinic and by the same surgeon. Measurements were performed in timely intervals according to the clinical needs of the patient. Non-shunted patients were seen every 4 weeks in order to perform the diagnostic CSF subtraction test with additional subsequent measurements. Shunted patients were seen every 2 weeks and were possibly extended to every 3 months if further valve adjustments became less likely necessary. Regular controls in stable patients were performed every 6 months. During measurements, it must be considered that the pressure sensor is located at the level of the skull surface and not intracranially, which might cause pressure deviations in the upright position.

### Patient cohort

In this study, 17 patients were included, who had either received a SR integrated into a CSF diverting shunt for diagnosing over- or under-drainage and adjusting the valve system guided by objective ICP data changes, accordingly (*n* = 11). In the remaining 6 patients, a stand-alone SR was placed in order to diagnose the ICP condition to clarify the possible indication for shunt treatment in uncertain CSF dynamic pathologies. Four out of six patients of the latter group underwent subsequent shunt placement due to acquisition of pathologic ICP measurements. Two of those patients were also measured as shunted patients, accordingly. Among the shunted patients, 5 suffered from idiopathic intracranial hypertension, 3 had a myelomeningocele and Chiari malformation type 2, one an aqueductal stenosis, 2 suffered from posthemorrhagic hydrocephalus due to prematurity, one from a MCM syndrome (megalencephaly-capillary malformation syndrome), and one patient had a craniopharyngioma. In the non-shunted population, the SR was implanted as a stand-alone reservoir to evaluate a possible idiopathic intracranial hypertension in 4 cases. One patient each had CSF dynamic disturbances due to a craniosynostosis or the aforementioned MCM syndrome, respectively. The median age in the shunted group was 15.8 years (range 4–35.2 years) and in the stand-alone group, 11.9 years (range 3.6–17.7 years; Table 1).

**Table 1:** Patient characteristics

**Characteristics**		**Shunted patients**	**Stand-alone patients**	***P*-value**
**Number**		11	6	
**Age (years, median)**		15.8 (range 4.0-35.2)	11.9 (range 3.6-17.2)	0.2
**Sex (F:M)**		9:2	4:2	
**BMI**		24.5 (range 12.4-43.2)	20.1 (range 14-29.7)	0.3
**Punctures (mean)**		-	3 (range 3-4)	
**Primarily with stand alone**		*2*	-	
**Secondary shunted**		-	*4 *	
**Shunt duration (months, median)**		80.1 (range 16-294)	10.7 (range 3-24)	
**Diagnosis**	**IIH**	4	4	
	**PHH**	2		
	**MMC**	3		
	**MCM**		1	
	**Craniosynostosis**		1	
	**Tumor**	1		
	**Aqueductal stenosis**	1		

*BMI* body mass index, *IIH* idiopathic intracranial hypertension, *PHH* posthemorrhagic hydrocephalus, *MMC* myelomeningocele, *MCM* megalencephaly-capillary malformation syndrome

### Data processing

The ICP data sampling was enabled by the SR reader unit. Therefore, a trowel, which is cable connected with the reader, was held directly over the skin where the SR is placed subcutaneously. A SD card saved the collected data in the reader and was used to transfer the anonymized data through a converter on a desktop computer as CSV files. After renaming the files in order to distinguish the different maneuver data, the data was imported into the ICP analysis software by drag and drop (https://icpicture.miethke.com/login.html; Miethke, Germany). The software is able to extract ICP AMP values from the acquired pulse pressure curves from a 44 Hz frequency data acquisition. Chart analysis was enabled in ICPicture software accordingly (Fig. 1). For further graphical and statistical elaboration, the collected data was exported and excel files to be copied into Prism (GraphPad, USA). In selected patients, according to the clinical judgment of the treating surgeon, a pressure relief was allowed by puncturing the reservoir and collecting about 10–15 ml of CSF under sterile conditions. That was performed in stand-alone-SR patients when ICP values were considered elevated (lying > 15 cmH_2_O, standing > 0 cmH_2_O). Clinical symptoms were evaluated after CSF puncture, and another ICP measurement was performed to verify ICP reduction. If patients had elevated ICP values and did show ameliorated clinical symptoms as well as lowering ICP values at least after three CSF punctures, the indication for shunt implantation was further discussed with the family. All measurements were carried-out between May 2020 and February 2022, and all but one subject in this study were investigated in an outpatient regimen.

#### Statistical analysis

Values are given as median with range or as mean and standard deviation as indicated, respectively. For comparisons of ICP and AMP measures between the two groups of patients an unpaired t-test was used, while differences within the groups among different maneuvers were evaluated with paired t-test evaluation. Linear regression was applied to extrapolate the correlations using the Pearson correlation coefficient among the analyzed parameters. Values of *p* < 0.05 were considered statistically significant. Statistical analysis was performed using Prism 9 software (GraphPad, San Diego, CA, USA) and after additional statistician consultation.

## Results

All patients could be measured according to the protocol, except for some of the Valsalva measurements in three patients (all aged < 6 years), which were only inconsistently completed. A total of 480 measurements were conducted. Most baseline characteristics showed no relation with the acquired measurements, beside analyzing BMI and ICP values, which showed a significant correlation in − 10° lying position in shunted patients.

**Fig. 2:**
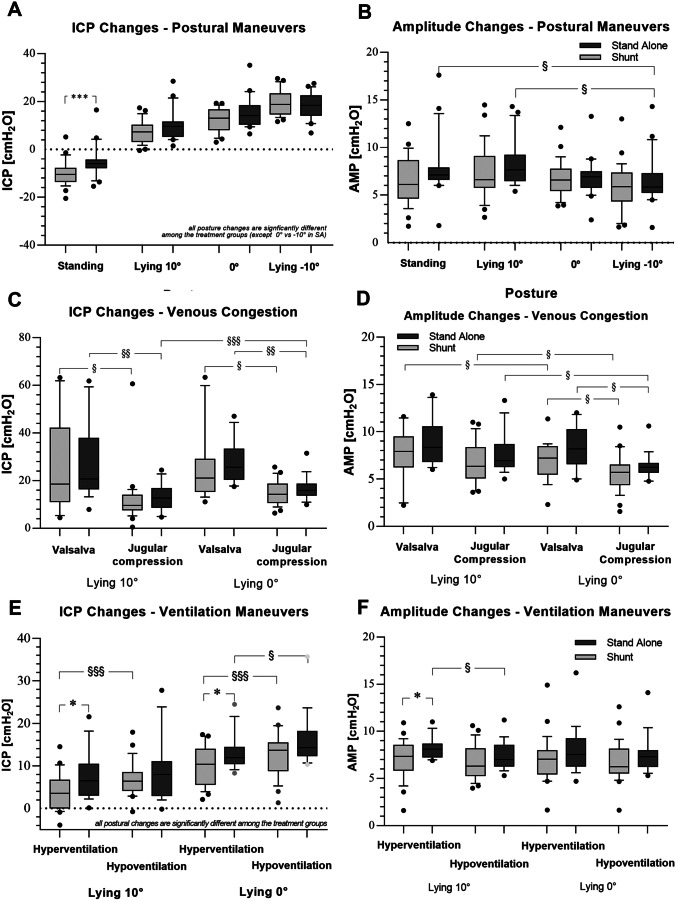
Boxplot diagrams visualizing the measured ICP and AMP values in the patient cohort of shunted and non-shunted patients acquired during the different maneuvers, respectively. The postural maneuvers resulted in significant ICP variations (**A**) and limited alterations in AMP (**B**). The venous congestion maneuvers showed a relevant increase in ICP, specifically in the Valsalva maneuver (**C**), and some significant changes in the pulse amplitude (**D**). The ventilation maneuvers caused significant changes between the groups, showing lower values during hyperventilation in shunted patients in 10° lying position (**E**), while amplitude changes were less pronounced (**F**)

The mean values of ICP and AMP are given in Table 2 and were visualized in Fig. 2. In general, ICP values showed more obvious changes in the different maneuvers in both groups compared to AMP changes. ICP measurements showed variations during the maneuvers in the range between –10 ± 5.1 and 25.1 ± 19.3 cmH_2_O in shunted and –5 ± 6.6 and 27.7 ± 15.8 cmH_2_O in non-shunted individuals; AMP values ranged from 5.8 ± 1.9 and 7.7 ± 2.6 in shunted and 6.9 ± 2.1 and 8.9 ± 2.4 in stand-alone patients. The postural maneuvers showed significant changes in ICP compared to other maneuvers in both groups. In particular, postural maneuvers in shunted patients caused significant differences in all body positions (standing: –10 ± 5.1 cmH_2_O; lying 10°: 7.4 ± 4.7 cmH_2_O; lying 0°: 12.4 ± 4.7 cmH_2_O; lying –10°: 19.5 ± 5.0 cmH_2_O; Fig. 2A). In the stand-alone-SR group, statistically significant changes in ICP were seen in all positions with the exception between the lying 0° and –10°, which may be due to the lower amount of individuals and measurements (standing: –5.0 ± 6.6c mH_2_O; lying 10°: 10.2 ± 6.2 cmH_2_O; lying 0°: 15.3 ± 6.5 cmH_2_O; lying –10°: 18.2 ± 5.3 cmH_2_O). Significant AMP differences due to postural changes were only seen in the stand-alone-SR patients. Specifically, the standing (7.7 ± 3.1 cmH_2_O) compared to –10° lying position (6.5 ± 2.6 cmH_2_O) and the 10° (8.3 ± 2.4 cmH_2_O) compared to –10° lying position (6.5 ± 2.6 cmH_2_O; Fig. 2B).

**Table 2:** Measurement values of ICP and AMP are given for shunted patients and stand-alone patients

**Position**	**Maneuvers**	**Shunted patients**			**Stand-alone patients**		
		**ICP**	**Amplitude**	***N***	**ICP**	**Amplitude**	***N***
**Standing**		− °10 ± 5.1^b^	6.5 ± 2.4	29	− °5.0 ± 6.61^a,b^	7.7 ± 3.1^c^	20
**Lying 10°**		7.4 ± 4.7^b^	7.3 ± 2.8	28	10.2 ± 6.2^b^	8.3 ± 2.4^c^	20
	***Hyperventilation***	3.7 ± 4.2^b,i^	7.1 ± 2.0	26	7.5 ± 5.6^a,b^	8.2 ± 1.1^a,i^	18
	***Hypoventilation***	6.8 ± 3.9^b^	6.7 ± 1.8	26	8.9 ± 7.2^b^	7.5 ± 1.5	19
	***Valsalva***	25.9 ± 19.3	7.7 ± 2.6	18	27.7 ± 15.8	8.9 ± 2.4	16
	***Jugular Compression***	12.0 ± 10.7^f^	6.8 ± 2.1	26	12.9 ± 5.7^d,h^	7.7 ± 2.2	18
**Lying 0°**		12.4 ± 4.7^b^	6.8 ± 1.9	29	15.3 ± 6.5^b^	6.9 ± 2.1	20
	***Hyperventilation***	9.9 ± 4.6^b,i^	7.1 ± 2.3	27	13.2 ± 4.2^a,b,i^	7.9 ± 2.5	18
	***Hypoventilation***	12.8 ± 5.0^b^	6.7 ± 2.1	27	15.9 ± 5.9^b^	7.6 ± 2.0	17
	***Valsalva***	25.3 ± 14.9^g,h^	6.9 ± 2.0	19	27.6 ± 8.7^e,h^	8.1 ± 2.2	15
	***Jugular Compression***	15.1 ± 5.0^f,g^	5.8 ± 1.9	27	16.6 ± 5.0^d^	6.4 ± 1.3	18
**Lying − 10°**		19.5 ± 5.0^b^	5.9 ± 2.4	26	18.2 ± 5.3^b^	6.5 ± 2.6	20

^a^ versus shunted

^b^ versus other body position within the same group (except 0° vs 10° in stand alone group without additional maneuvers)

^c^ versus lying − °10°

^d^ versus jug compression 10°

^e^ versus jug compression 0°

^f^ versus Valsalva in same body position

^g^ versus 10° during same maneuver

^h^ versus jug compression 0°

^i^ versus hypoventilation in same body position.

Mild venous congestion maneuvers showed significant ICP differences between jugular compression (lying 0° shunted: 15.1 ± 5.0 cmH_2_O; stand-alone: 16.6 ± 5.0 cmH_2_O) and Valsalva (lying 0° shunted: 25.3 ± 14.9 cmH_2_O; stand-alone: 27.6 ± 8.7 cmH_2_O) in both groups and in both lying positions with higher values caused by the Valsalva maneuver, respectively (Fig. 2C). In general, the highest ICP values could be reached during Valsalva maneuvers in both lying positions (lying 10° shunted: 25.9 ± 19.3 cmH_2_O; stand-alone: 27.7 ± 15.8 cmH_2_O) and were provoked by the patients themselves. Significant AMP differences were seen in shunted patients during Valsalva and jugular vein compression between 10° (shunted Valsalva: 7.7 ± 2.6 cmH_2_O; jug. compression: 6.8 ± 2.1 cmH_2_O) and 0° (shunted Valsalva: 6.9 ± 2.0 cmH_2_O; jug. compression: 5.8 ± 1.9 cmH_2_O) as well as between Valsalva and jugular vein compression at 0° lying position. In stand-alone SR patients, significant changes were seen during jugular compression between 10° (8.3 ± 2.4 cmH_2_O) and 0° lying position (6.9 ± 2.1 cmH_2_O) and also between Valsalva and jugular vein compression at 0° lying position (Valsalva: 8.1 ± 2.2 cmH_2_O; jug. compression: 6.4 ± 1.3 cmH_2_O; Fig. 2D).

In ventilation maneuvers, ICP significantly changed in the shunted population with lower values in hyperventilation compared to hypoventilation, as seen in 10° (hyperventilation: 3.7 ± 4.2 cmH_2_O; hypoventilation: 6.8 ± 3.9 cmH_2_O) as well as in 0° lying position (hyperventilation: 9.9 ± 4.6 cmH_2_O; hypoventilation: 12.8 ± 5.0 cmH_2_O). In non-shunted patients, a similar change was also detectable, but only in the 0° lying position (hyperventilation: 13.2 ± 4.2 cmH_2_O; hypoventilation: 15.9 ± 5.9 cmH_2_O). In addition, stand-alone-SR patients showed higher ICP values during hyperventilation compared to shunted patients in both lying body positions (shunted 10°: 3.7 ± 4.2 cmH_2_O; stand-alone 10°: 7.5 ± 5.6 cmH_2_O; shunted 0°: 9.9 ± 4.6 cmH_2_O; stand-alone 0°: 13.2 ± 4.2 cmH_2_O; Fig. 2E). AMP changes were less relevant. Significant differences were seen, however, in the stand-alone-SR group for the hyperventilation compared to hypoventilation in 10° lying position (hyperventilation: 8.2 ± 1.1 cmH_2_O; hypoventilation: 7.5 ± 1.5 cmH_2_O) and between the two groups during hyperventilation in 10° lying position (shunted: 7.1 ± 2.0; stand-alone: 8.2 ± 1.1 cmH_2_O; Fig. 2F).

The correlation analysis between ICP and AMP values, as a possible indicator for impaired compliance, showed a significant relation in the stand-alone-SR patient group. Specifically, Valsalva and jugular vein compression showed significant correlations in both measured lying body positions (Valsalva 0°: *r*^2^ 0.674, 3.2 (1.9–4.6); 10°: *r*^2^ 0.777, 5.7 (3.9–7.5); jug. compression 0°: *r*^2^ 0.467, 2.7 (1.1–4.2); 10°: *r*^2^ 0.568, 2.0 (1.1–2.9); *p* < 0.01; Table 3). During hyperventilation, a correlation was also found but only in the 10° body position (*r*^2^ 0.51, 3.5 (1.7–5.3); *p* < 0.01). In shunted patients, a significant correlation between ICP and AMP was only found during hyperventilation in both lying body positions (0°: *r*^2^ 0.153, 0.8 (0.02–1.5); 10°: *r*^2^ 0.165, 0.8 (0.04–1.6); *p* < 0.05; Table 3).

**Table 3:** Correlation analysis between ICP and AMP during venous congestion and ventilation maneuvers

**Stand alone**	**Patients (n)**	**Maneuvers (n)**	**R**^2^	**P**	**Slope (95% CI)**
Valsalva 0°	6	14	0.674	** < 0.001**	3.2 (1.9–4.6)
Valsalva 10°	6	15	0.777	** < 0.001**	5.7 (3.9–7.5)
Jugular compression 0°	6	17	0.467	**0.002**	2.7 (1.1–4.2)
Jugular compression 10°	6	17	0.568	** < 0.001**	2.0 (1.1–2.9)
Hyperventilation 0°	6	17	0.024	0.54	0.6 (− 0.6 to 1.1)
Hyperventilation 10°	6	17	0.51	**0.001**	3.5 (1.7–5.3)
**Shunted**			**R**^**2**^	**P**	**Slope (95% CI)**
Valsalva 0°	13	13	0.04	0.4	1.5 (− 2.1 to 5.2)
Valsalva 10°	13	13	0.0003	0.9	0.1 (− 3.8 to 4.1)
Jugular compression 0°	13	21	0.04	0.3	0.5 (− 0.5 to 1.5)
Jugular compression 10°	13	20	0.08	0.2	1.5 (− 0.6 to 3.6)
Hyperventilation 0°	13	21	0.153	**0.04**	0.8 (0.02–1.5)
Hyperventilation 10°	13	20	0.165	**0.04**	0.8 (0.04–1.6)

### Representative case description

**Fig. 3:**
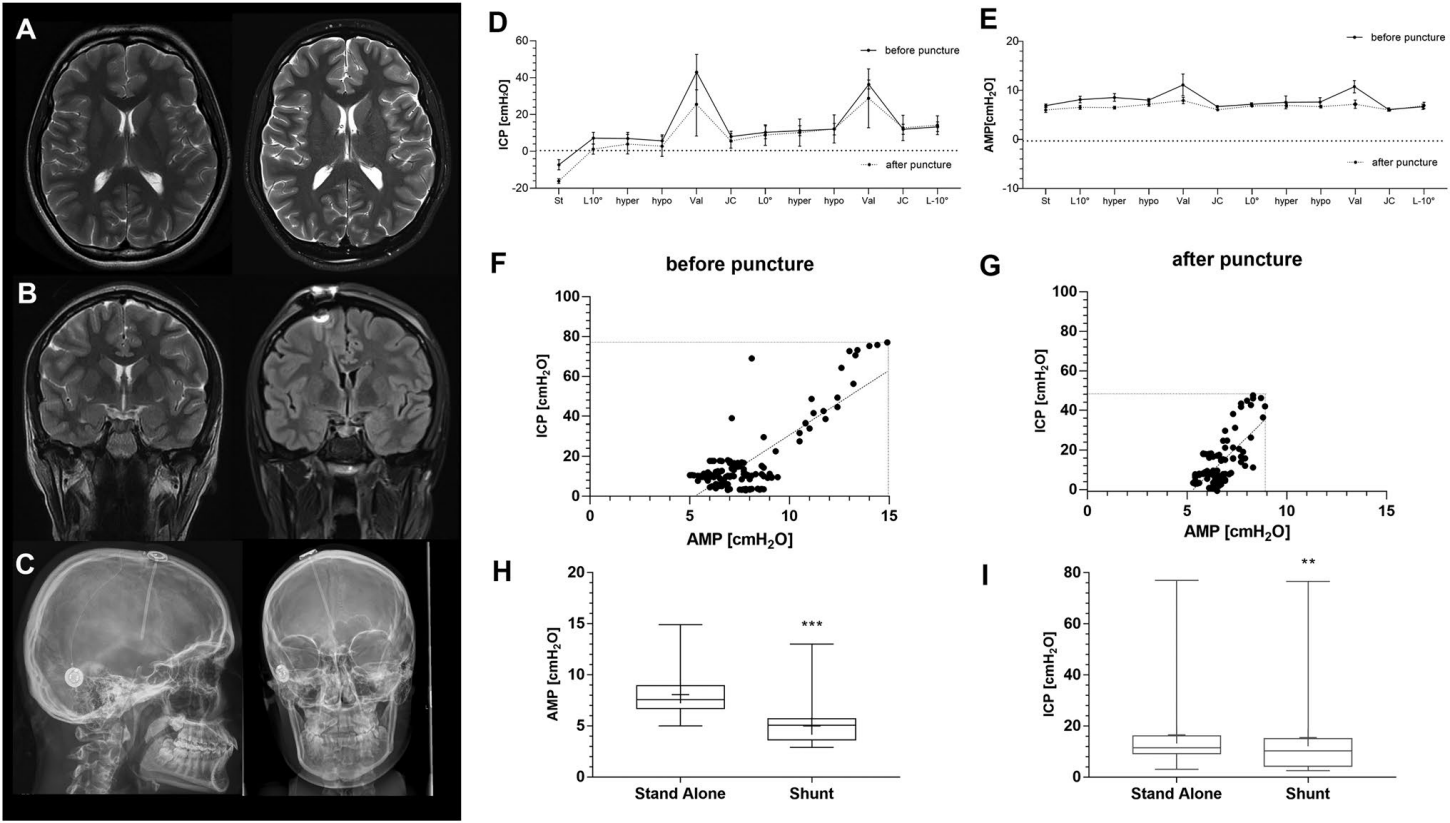
Representative case example of a 17-years-old female patient with chronic headache and visual field impairment without papilledema in fundoscopy. Lumbar puncture revealed a high opening pressure of > 30 cmH_2_O. High doses of acetazolamide treatment were associated with treatment-related side effects. The further diagnostic evaluation was planned by using a stand-alone sensor reservoir (SR). The ventricular catheter was placed as a guided procedure in the narrow right frontal horn of the lateral ventricle [16, 17] connected to the SR (**A**, **B**). ICP measurements confirmed high pressure and amplitude values, which showed reduction after CSF puncture from the reservoir (~ 10 ml) (**D**, **E**) in parallel with relief of headaches and subjective amelioration of vision. In addition to a significant reduction of ICP after CSF tapping, also AMP values were reduced together with a gradual decrease of the Pearson correlation index (0.69 (0.58–0.78) to 0.66 (0.59–0.72; **F**, **G**). Strikingly, the upper AMP values as well as ICP values completely disappeared after volume relieve (**F**, **G**). Thus, maneuver-aided tICPM and follow-up confirmed the indication for ventriculo-peritoneal shunting using an M.blue valve at 15/ + 30 (15/45) cmH2O opening pressure (**C**). Comparing the mean measured values throughout the maneuver protocol before and after shunting the ICP and AMP values proved significant reduction (**H**, **I**)

A 17-years-old female patient with a long history of headache and visual field cut (scotoma) was presenting with unclear condition of a suspected idiopathic intracranial hypertension (IIH) showing high opening pressure of > 30 cmH_2_O twice in lumbar puncture, however, without papilledema in fundoscopy and missing secondary signs in MRI such as empty sella or venous sinus obstruction. She was treated elsewhere with high doses of acetazolamide (1.5 g/d), which led to a limited benefit of the visual field cuts. However, with additional treatment-related side effects of hypokalemia and nausea. In addition, psychological issues such as chronic history of attention deficit hyperactivity disorder made the therapeutic conclusion even more chalenging. Thus, further diagnostic information was needed before possible shunt implantation, and a stand-alone SR was implanted for ICP measurements during maneuver protocol. Surgery was accomplished uneventfully by placing the ventricular catheter as a guided procedure in a narrow right frontal horn of the lateral ventricle [16, 17]. The catheter was connected to the SR at the burrhole level and the side opening of the SR was closed with a blind connector fixed to a short silicone catheter. The patient was followed up and measured regularly for 3 months. ICP measurements confirmed high ICP and AMP values during the different maneuvers. After CSF withdrawal (~ 10 ml), transient relief of headaches, as well as subjective amelioration of vision, was reported. In parallel, ICP as well as AMP values were significantly reduced (Fig. 3A/B). The Pearson correlation quotient dropped from 0.69 (0.58–0.78) to 0.66 (0.59–0.72) after transient CSF relieve. Following 3 months of maneuver-aided tICPM and three reservoir tapings, the results confirmed increased ICP values with impaired compliance and amelioration after CSF tapping, thus, indicating a ventriculo-peritoneal shunt implantation. The shunt was implanted using an M.blue valve at 15/ + 30 (15/45) cmH2O opening pressure, connecting the shunt to the preexisting sensor reservoir (Fig. 3C–E). Acetazolamide was gradually reduced and finally stopped. The patient remained on a regular follow-up (every 3–6 months), describing a continuous better control of headaches and amelioration in vision according to ophthalmology consultation. Comparing the mean measured values throughout the maneuver protocol before and after shunting, the ICP values, as well as AMP values, were reduced significantly (Fig. 3F–H).

## Discussion

Hydrocephalus remains a complex disease and comes with ongoing challenges in terms of treatment decisions and shunt adaptations, especially in individual complicated cases [18]. Telemetric ICP measurements in shunted patients as well as in patients with unclear indications for possible shunting reveal a new perspective of theranostic option for hydrocephalus treatment. The most effective measurement algorithm and analysis of tICPM for clinical routine implementation remain unproven. The introduction of a maneuver protocol to initiate a wide range of ICP conditions as suggested in the present study allowed a systematic analysis not only of the ICP but also of AMP changes. The maneuver protocol includes different body positions inducing changing conditions of intracranial CSF and venous outflow, while Valsalva and jugular compression maneuvers caused mild venous congestion, and finally, ventilation maneuvers provoke altering conditions of vascular reactivity, and thereby changing the intracranial blood volume. In the present study, we were able to observe most significant differences of ICP value changes in the postural maneuvers, which were similar among shunted and non-shunted patients. However, in standing position the shunted patients showed lower values. Highest ICP values were shown in venous congestion maneuvers, specifically during Valsalva maneuver. Hypoventilation showed higher ICP values compared to hyperventilation, while there were differences among the groups in both ventilation maneuvers in 10° lying position. AMP changes in postural maneuvers were only seen in non-shunted patients. However, in combination with venous congestion AMP, changes could also be observed in shunted patients. Changes in AMP during ventilation maneuvers could only be seen in 10° lying position in stand-alone patients and also differences between the groups during hyperventilation.

New insights from telemetric intracranial pressure monitoring in shunted patients may lead generally to the following conclusions: Negative ICP values in standing positions are considered to be physiologic [10, 19] and shunted patients may show more negative ICP values than non-shunted patients [4, 20]. Shunt adjustments do result in changes of intracranial pressure values [4, 6, 21]. However, shunted patients may encounter variable individual normal range of ICP values correlating differently with clinical status [6, 8]. The interpretation of ICP values together with AMP changes may be relevant for better understanding of individual pathophysiological conditions of impaired intracranial compliance [12, 19, 20]. In addition, it was reported that objectifying shunt adjustments by using tICPM, especially in clinically difficult cases, is advantageous for evaluating the potential of improving the patients condition [6].

Nevertheless, tICPM is still a cumbersome method in terms of data management and data interpretation. Thus, we elaborated maneuvers, which can be implemented in an outpatient setting to provoke a wide range of ICP changes and analyze the alterations as well as the correlating ICP and AMP changes, accordingly. Our measurement protocol of body position changes was selected to provoke obvious alterations between standing and lying position and include subtle changes from positive 10° to negative 10°, in which jugular vein collapse can be excluded. The vast majority of maneuvers could be easily applied to all our patients. Ventilation maneuvers, as well as mild venous congestion, do need exessive cooperation from pediatric patients. From our experience, only the Valsalva maneuver appears to be more challenging and was only inconstantly applicable in patients younger than 6 years of age. However, it was very valuable since the highest ICP changes could thereby be observed.

Our measurements revealed wide ranges of ICP changes, which were partially correlating with AMP changes. This is in accordance with the literature, even though no study so far had directly compared the two categories of shunted and none shunted patients in the pediatric age group [11, 12]. We observed that position-dependent maneuvers generate the most significant ICP changes, as confirmed by previous authors [8, 9]. Concerning posture maneuver-dependent AMP changes, less pronounced or even no variations were reported. Methodologically, it was feasible to automatically detect the AMP changes offline by using the recently developed software ICPicture (Miethke Co., Potsdam, Germany), which appears to be a simple and user-friendly ICP analysis software. Another very valuable software tool such as ICM + (Cambridge, UK) was introduced by other authors in overnight ICP monitoring and shunt infusion studies in which amplitude analysis and other parameters are integrated [14, 19, 22], but seems to be more complex and less user friendly as it is mainly used as a more sophisticated research tool.

Pathophysiologic intracranial pressure conditions are associated with neurocognitive developmental disturbances. The relation between intracranial volume and ICP changes is given as intracranial compliance. Disturbed compliance is present if small volume changes lead to a high ICP increase. It remains a challenge to measure intracranial compliance by noninvasive means. Previous reports have emphasized the value of determining amplitude changes in the intracranial pressure curve, specifically if amplitude value increases relevantly at high ICP values [23–25]. It was additionally described that periodically detected correlation between both values as Pearson coefficient index (RAP) during long-term ICP monitoring may give decent information on possible disturbances in intracranial compliance [24]. In contrast to intensive care ICP monitoring, telemetric ICP monitoring detects relatively short periods. The advantage of using a maneuver protocol in tICPM enables the detection of a wide range of ICP changes in a reasonable amount of time, in which amplitude changes may also well be recognized. The current feasibility study should give first insights for the maneuver protocol during tICPM detecting ICP and AMP values with first implications of its potential for intracranial compliance evaluation without the need of an infusion tests.

As it was already demonstrated in previous studies, pulse amplitude is not sensibly affected by positional shifts [9, 12, 15], also confirming that patients were measured with intact compliance according to the pressure–volume curve. Pulse amplitude was previously described to be an indicator of cerebral compliance [20]. However, the pulse wave amplitude is relevantly determined by the cardiac stroke volume and the cerebrovascular resistance, which is also influenced by body posture changes [12], and makes any interpretation toward intracranial compliance during these maneuvers more difficult. That seems to be less relevant for venous congestion maneuvers, in which no direct influence on cardiac output is induced. As already stated, the Valsalva maneuver resulted in the most effective way to induce a significant increase of the ICP, but Jugular vein compression had the advantage, that it did not depent as much on patient cooperation. To our knowledge, this is the first investigation of variations of ICP in shunted and non-shunted pediatric patients using mild venous congestion maneuvers. Both jugular compression and Valsalva maneuvers showed a clear correlation between ICP and AMP changes. Thus, venous congestion maneuvers are very valuable to analyze possible changes in ICP and AMP and to draw conclusions on possible impaired intracranial compliance.

In the present study, we have applied the maneuver protocol also in patients for diagnostic evaluation of ICP changes with a so-called stand-alone SR to evaluate the indication for possible shunt implantation. Interestingly, we observed in the stand-alone SR group similar but more pronounced ICP changes but also statistically significant changes in AMP, which were also correlating with ICP measures (Table 3). A recent large study on an adult population detected significant relation between postural and day/night changes of ICP and pulse amplitude values in shunted and non-shunted individuals [19]. Similarly, in NPH patients, a significant reduction of ICP and ICP pulse amplitude was observed after shunting toward a normal level [20]. Shunt adjustment-related changes were described in ICP as well as ICP amplitude changes compared to a ligated shunt system and did further show reduction after walking activity [15]. In the present study, we were able to observe in the stand-alone SR group even more sensitive maneuver-related changes for ICP and AMP changes. These observations are described for the first time for ventilation and mild venous congestion maneuvers in a cohort of pediatric-originating CSF disturbances. Although the evaluated population remains to be limited in terms of number, age, and pathology variations, the data is promising and seems to discover a huge potential for diagnosing unclear cases of CSF dynamic disturbances. The suggested protocol might thus be performed on an individual basis after implantation of a stand-alone SR, applying maneuver-related measurements and interpreting ICP as well as AMP changes as well as their correlation for clarifying the condition of intracranial compliance. In addition, CSF punctures can be applied to investigate symptom changes after CSF relief, which might further be objectified by a repeated tICPM maneuver protocol and observing possible ameliorations of ICPs as well as AMP patterns, finally enabling a solid decision-making in terms of shunt indication.

Our case example is representing this approach on an individual level. The patient showed untypical patterns in MR imaging for IIH and had a lack of papilledema. However, headaches, visual field cuts, and increased LP opening pressures were indicating IIH. We suggested performing stand-alone SR measurements applying the maneuver protocol, which confirmed an increase in ICP, as well as in ICP amplitude. Both were positively influenced by CSF relieve performed by reservoir puncture. After indicating shunt therapy, significant ICP as well as ICP amplitude value reduction are observed (Fig. 3). In fact, amplitude values were measured at 7.6 ± 1.9 cmH_2_O (range 5–15) before and 6.6 ± 0.8 cmH_2_O (range: 5–9) following CSF relief by tapping, as well as at 8 ± 1.9 cmH_2_O (5–15) before and 5 ± 1.6 cmH_2_O (3–13) after shunting ameliorating intracranial pressure changes during the cardiac cycle as a sign for improved intracranial compliance after CSF drainage. However, the correlation coefficient between both values after tapping was reduced less strikingly from 0.69 (0.58–0.78) to 0.66 (0.59–0.72). In contrast to long-term ICP monitoring, it might be discussed that the correlation coefficient may not serve as a reliable parameter for intracranial compliance in tICPM. However, the decrease in amplitude values together with the decrease in ICP may well identify the amelioration of intracranial compliance before and after CSF relief as measured in a wide range of ICP measurements during the maneuver protocol. Specifically, the venous congestion, as well as ventilation maneuvers, did show the relation of ICP and AMP changes, indicating a lack of intracranial compliance in the stand-alone group, which obviously improved after shunting. Finally, the clinical amelioration after reservoir puncture and shunt implantation confirmed the relevance of the tICPM in the representative case.

## Conclusions

The SR offers a feasible tool to evaluate ICP characteristics on an outpatient basis in complex hydrocephalus cases. The implementation of the related software for the elaboration of ICP as well as ICP amplitude changes provides the opportunity to simply elaborate relevant parameters in different conditions. Our maneuver protocol offers the possibility to investigate a wide range of ICP changes applicable in the outpatient clinic. Possible correlations of increased ICP with AMP changes may indicate impaired intracranial compliance. We conclude that body posture changes in standing and lying positions together with mild venous congestion in lying position, specifically jugular vein compression, added by possible reservoir CSF puncture, maybe the simplified and relevant protocol for outpatient tICPM. This will be easily applicable to all patients with a reasonable amount of time and effort. We observed to receive relevant results from our measurements and postulate that future investigations might concentrate on these kinds of maneuver protocols to possibly avoid cumbersome efforts of long-term ICP measurements, overnight studies, or infusion tests. Further investigations are required to assess the reliability of the proposed measures on a bigger amount of patients with variable underlying pathologies.
